# MRI scanner‐independent specific absorption rate measurements using diffusion coefficients

**DOI:** 10.1002/acm2.12095

**Published:** 2017-05-04

**Authors:** Youngseob Seo, Zhiyue J. Wang

**Affiliations:** ^1^ Medical Metrology Center Korea Research Institute of Standards and Science Yuseong‐Gu Daejeon 34113 Republic of Korea; ^2^ Department of Radiology University of Texas Southwestern Medical Center and Children's Medical Center Dallas Dallas TX 75390 USA

**Keywords:** diffusion coefficient, diffusion tensor imaging, human torso phantom, MR safety, specific absorption rate

## Abstract

**Objective:**

The purpose of this study was to measure specific absorption rate (SAR) during MRI scanning using a human torso phantom through quantification of diffusion coefficients independently of those reported by the scanner software for five 1.5 and 3 T clinical MRI systems from different vendors.

**Methods:**

A quadrature body coil transmitted the RF power and a body array coil received the signals. With diffusion tensor imaging, SAR values for three MRI sequences were measured on the five scanners and compared to the nominal values calculated by the scanners.

**Results:**

For the GE 1.5 T MRI system, the MRI scanner‐reported SAR value was 1.58 W kg^‐1^ and the measured SAR value was 1.38 W kg^‐1^. For the Philips 1.5 T MRI scanner, the MRI system‐reported SAR value was 1.48 W kg^‐1^ and the measured value was 1.39 W kg^‐1^. For the Siemens 3 T MRI system, the reported SAR value was 2.5 W kg^‐1^ and the measured SAR value was 1.96 W kg^‐1^. For two Philips 3 T MRI scanners, the reported SAR values were 1.5 W kg^‐1^ and the measured values were 1.94 and 1.96 W kg^‐1^. The percentage differences between the measured and reported SAR values on the GE 1.5 T, Philips 1.5 T, Siemens 3 T, and Philips 3 T were 13.5, 6.3, 24.2, 25.6, and 26.6% respectively.

**Conclusion:**

The scanner‐independent SAR measurements using diffusion coefficients described in this study can play a significant role in estimating accurate SAR values as a standardized method.

## 1 Introduction

Magnetic resonance imaging (MRI) generates images for medical diagnoses of diseases using a static magnetic field and time‐varying electromagnetic fields generated by a radiofrequency (RF) transmit coil and x‐, y‐, and z‐gradient coils, respectively. These time‐variant electromagnetic fields induce electric currents and voltages in the conductive human body when positioned inside an MRI scanner.

The eddy currents induced by the time‐varying electromagnetic fields during an MRI scan can cause undesired heating of patients due to the deposition of RF power into the body, and this is a significant safety concern.^1^, ^2^, ^3^, ^4^, ^5^, ^6^, ^7^, ^8^, ^9^, ^10^, ^11^ It is thus necessary to determine the RF energy absorbed by the body in terms of the specific absorption rate (SAR). According to International Electrotechical Commission (IEC), the SAR value should be limited to 3.2 W kg^‐1^ for the head and 4.0 W kg^‐1^ for body applications for durations of 6 min.^12^ Similarly, the Food and Drug Administration (FDA) of the United States requires that the SAR should be less than 4 W kg^‐1^ when averaged over the entire body for 15 min and 3 W kg^‐1^ for the head for 10 min.^13^ The risk of hyperthermic tissue damage is relatively serious for neonates and for children who cannot communicate verbally, as well as for patients who have insensate limbs and those who are under anesthesia during the MRI scan.

Commercial MRI scanners provide an estimated SAR level for each scan; this level is calculated from the RF waveforms and sequence parameters, system calibration, Q factors and loading of the RF transmit coil, etc. The SAR calculation assumes certain average parameters, which in reality can vary from scanner to scanner and may change over time.^14^ Incorrect manufacturer‐reported SAR values have been acknowledged for clinical MR imaging systems.^14^, ^15^, ^16^ For example, one study found a scanner overestimated the SAR by up to 2.2 folds.^15^ Even before the highest allowed SAR level has been reached, a patient's sweating during an MRI can raise concerns of possible overheating. On the other hand, overestimating the SAR can prevent certain important scans to be run on a patient. It is also conceivable that a malfunction in the quadrature RF transmit coil can generate RF with higher levels in the counter rotating component, resulting in higher than expected power deposition levels. Direct estimation of SAR values independent of the level calculated by MRI scanners is therefore desirable.

Numerical calculations of RF energy deposition levels have been performed to predict SAR levels in anatomical models consisting of homogeneous cylinders, spheres, or in head models.^1^, ^2^, ^3^, ^4^, ^17^, ^18^, ^19^, ^20^ There is a large range of variability in SAR levels for different pulse sequences. Global and local SAR measurements at different B_0_ magnetic field strengths and measurements of exposure to different RF coils have been conducted.^21^, ^22^, ^23^, ^24^ However, direct measurement of RF heating in a clinical setting has not been an easy task. Temperature measurement using optical thermometry only yields values in few spatial points. In the present study, we demonstrate the measurement of SAR of a human torso phantom using diffusion MRI in clinical MRI systems from different vendors.

## 2 Materials and methods

### 2.A MRI scanners

SAR levels were measured on five MRI scanners: an Achieva 1.5 T (Philips Healthcare), a Signa Excite 1.5 T (GE Healthcare), a Magnetom Verio 3 T (Siemens Healthcare), and two Achieva 3 T (Philips Healthcare) systems. SAR values for three different MRI sequences with SAR values (1.48, 1.5, 1.58, and 2.5 W kg^‐1^ nominal values as reported by MRI scanners) were measured on various scanners at two discrete magnetic field strengths. Image acquisition parameters are summarized in Table 1. The RF excitation power was transmitted by an integrated RF body coil (Multi‐transmit mode = “NO” at Philips 3 T). A four‐ or eight‐channel body array coil was employed as a receive coil in this study.

**Table 1:** Image acquisition parameters at 1.5 and 3.0 T

	Image sequence		
	T1w TSE	T1 TIRM	T2w TSE
TR/TE [ms]	800/10	1150/9.3	4710/110
TI [ms]	n/a	220	n/a
Field of view [mm^2^]	400 × 400	400 × 400	400 × 400
No. of slices	10	8	10
Slice thickness [mm]	6	6	6
Acquisition matrix	400 × 400	256 × 256	200 × 154 (reconstructed to 400 × 400)
Voxel size [mm^3^]	1 × 1 × 6	1.56 × 1.56 × 6	1 × 1 × 6
Slice orientation	Transverse	Transverse	Transverse
Phase‐encoding direction	AP	AP	AP
NSA	2	1	4
Total scan time (GE 1.5 T/ Philips 1.5 T)	5 min 12 s/4 min 56 s	4 min 18 s	4 min 23 s
Parallel imaging method	No	GRAPPA for Siemens 3 T	No
Bandwidth [Hz/pixel] (GE 1.5 T/ Philips 1.5 T)	260/290	260	334

TSE, Turbo spin echo; TIRM, Turbo inversion recovery magnitude.

### 2.B Human torso phantom morphology

**Figure 1:**
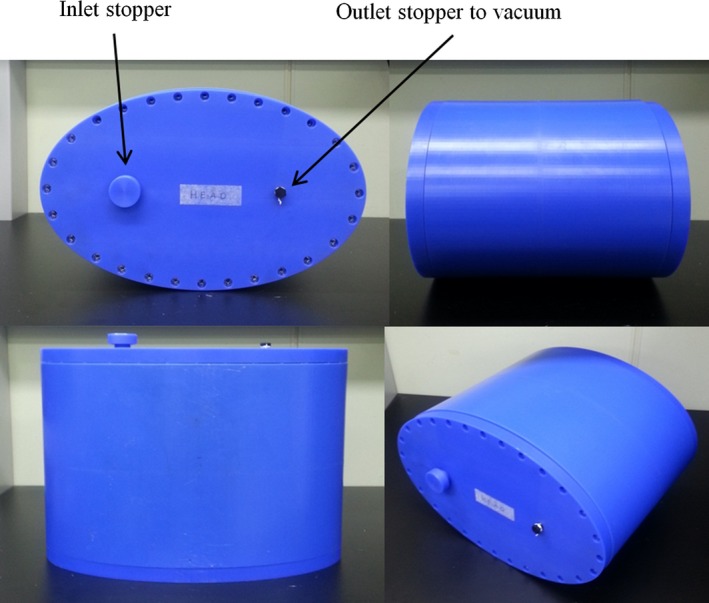
Phantom morphology mimics the shape of the human torso.

A cylinder‐shaped human torso phantom (50 cm (L) × 43 cm (W) × 28 cm (H)) was constructed on the basis of U.S. anthropometric reference data 25 (Fig. 1). The airtight plastic phantom container (15 mm thickness) was filled with a volume of approximately 16.6 L of a hydroxy‐ethyl cellulose (HEC) gelled‐saline solution consisting of 25.7 g of NaCl, 514.6 g of HEC powder, and 16.6 L of distilled water, simulating human tissue, as described in the American Society of Testing Materials (ASTM) International standard method for SAR measurements.^26^

The gel thermal properties (thermal diffusivity = 1.4 × 10^‐7^ m^2^s^‐1^ and heat capacity = 4156 J/(kg°C)) were measured with a thermal property analyzer (KD2, Decagon Devices Inc., Pullman, WA, USA). The electric conductivity (*σ* = 0.48 ± 0.04 S m^‐1^ at 64 MHz and 0.49 ± 0.04 S m^‐1^ at 128 MHz) and relative electric permittivity (*ε*_r_ = 76.48 ± 3.98 at 64 MHz and 76.22 ± 4.12 at 128 MHz) of the gel solution were measured using a dielectric assessment kit (DAK‐12, SPEAG Ltd., Zurich, Switzerland). A vacuum was created in the phantom container to eliminate air bubbles in the gel phantom**.**

### 2.C Independent SAR assessment using diffusion measurement

**Figure 2:**
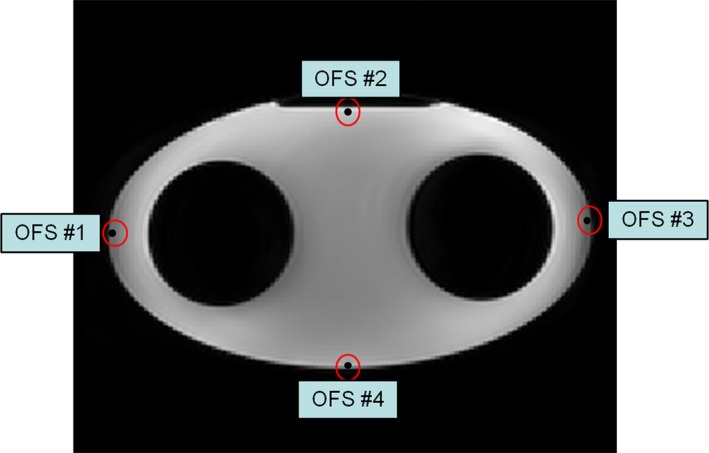
Location of four optic fiber temperature sensors in the phantom periphery, used to measure the initial temperatures of the phantom and the time taken to reach equilibrium with the environment.

Four optic fiber temperature sensors (OFS, Neoptix Inc., Quebec, Canada) were placed at the periphery of the gel phantom at 28°C to certify that there was minimal heat loss to the environment during the measurements (Fig. 2). We measured the initial temperatures of the sensors positioned in the phantom and the time it took to reach equilibrium with the environment. We considered that thermal equilibrium has been reached when the difference between temperatures measured by the sensors in the phantom and temperature inside the magnet bore was less than 0.1°C.

**Figure 3:**
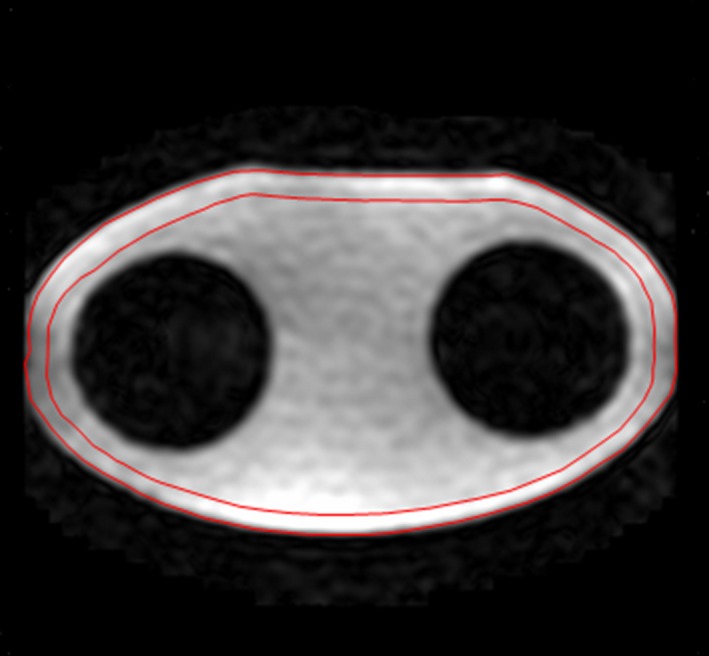
The apparent diffusion coefficient within an ROI (red) which was manually placed on the periphery of the phantom was quantified using a set of diffusion‐weighted images.

On each MRI scanner, the heating of the gel phantom caused by a high SAR sequence was assessed by the changes in the mean diffusivity (MD) value, before and after running the high SAR image sequence.^26^ A region‐of‐interest (ROI)‐based MD calculation was performed for the SAR measurements. In the ROI‐based quantification, the average signal intensity within the ROI as shown in Fig. 3 for the b = 0 image and each high b diffusion‐weighted image was measured first. Using these values, a diffusion tensor was calculated, and MD value was obtained. The water diffusion coefficient in the gel was practically identical to that of free water,^27^ and the diffusion coefficient (D) was very sensitive to the temperature (T).^28^, ^29^ The temperature was calculated using the following equation:^28^

(1) $$D=D_{0}\cdot {\left[\left(T/T_{s}\right)-1\right]}^{\gamma }$$

where D_0_ = 1.635 × 10^‐8^ m^2^ s^‐1^, T_s_ = 215.05 K, and *γ* = 2.063. The mean diffusivity measured by DTI is the same as the diffusion coefficient (D) in Eq. (1).

Verification of temperature changes obtained by the diffusion coefficients using Eq. (1) was performed by comparison to those measured by four optic fiber temperature sensors positioned as in Fig. 2. The mean diffusion coefficients within each ROI which were manually drawn around the temperature sensors were calculated using software written in IDL 8.4 (IDL Research Systems Inc., Boulder, CO, USA) before and after the high SAR image sequence at 3 T. The MD value was the average over 14 pixels within the ROI in one slice showing the tip of the sensors.

**Figure 4:**
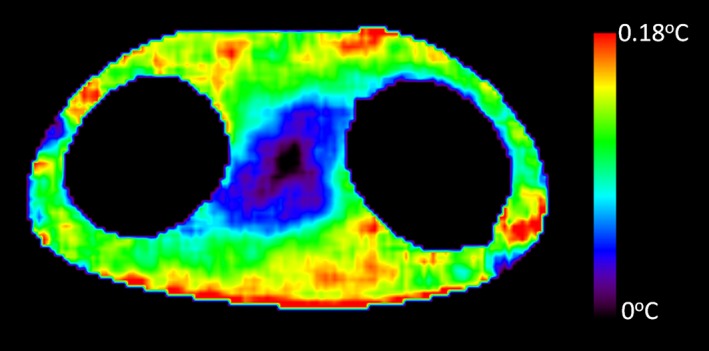
Temperature change derived from the difference between mean diffusivity (MD) maps is shown for one Philips 3T scanner.

For each study, the phantom was placed in the scanner room for at least 24 hr to establish thermal equilibrium with the environment. The same phantom weight (18 kg) was entered into the MRI system at registration. First, the SAR value induced by the diffusion tensor imaging (DTI) scan was measured for the torso phantom on each MRI scanner using repeated DTI scans. Second, in order to measure the SAR value caused by the high SAR sequences, an axial DTI scan was initially conducted, followed by several minutes of high SAR scan. The DTI scan was then repeated. The scanner‐specific DTI acquisition parameters are listed in Table 2. Auto‐shim was utilized in all studies. The mean diffusivity within a ROI which was manually placed at the periphery of the phantom was evaluated for each DTI scan using software written in IDL 8.4 (Fig. 3), and the temperature change derived from the difference between the MD maps was estimated (Fig. 4). The standard error of the mean diffusivity for the ROI ranges from 0.0005 × 10^‐3^ to 0.0006 × 10^‐3^ mm^2^ s^‐1^ for the 5 scanners based on repeated measurements.

**Table 2:** DTI acquisition parameters for each scanner

	GE 1.5 T	Philips 1.5 T	Siemens 3 T	Philips 3 T
b‐values [s mm^‐2^]	0, 1000	0, 1000	0, 1000	0, 1000
No. of gradient directions	30	30	30	30
TE [msec]	80	82	88	74
TR [msec]	9218	8000	7100	6844
FoV [mm^2^]	400 × 400	380 × 380	400 × 400	400 × 400
Acquisition matrix	128 × 128	128 × 128	128 × 128	128 × 128
Slice thickness [mm]	4	4	4	4
No. of slices	16	14	14	14
Parallel imaging method	ASSET	SENSE	GRAPPA	SENSE
Acquisition time	5 min 12 s	4 min 56 s	4 min 18 s	4 min 23 s

The SAR values and the temperature change are related according to the equation below^26^:

(2) $$C_{p}\Delta T={\text{SAR}}_{\mathrm{DTI}}\cdot {\text{TA}}_{\mathrm{DTI}}+{\text{SAR}}_{\mathrm{high}}\cdot {\text{TA}}_{\mathrm{high}}$$

Here, C_p_ (= 4.18 kJ/(kg°C)) is the specific heat of the phantom, ΔT is the temperature change in °C, and TA is the acquisition time of the pulse sequence. The first term on the right‐hand side is the heating from the DTI sequence, and the second term on the same side represents the heating from the high SAR sequence under investigation. One SAR measurement was performed in one MRI session, and then the phantom was placed in the scanner room for one day to reach thermal equilibrium with the environment. SAR measurements were repeated at least 10 times for each MRI scanner and the mean and standard deviation (SD) were calculated. Percentage differences between the measured mean and reported SAR values on the MRI scanners were calculated.

## 3 Results

The results of the temperatures measured by the optic fiber sensors positioned at the phantom periphery and the times taken to reach equilibrium with the environment are summarized in Table 3. At initial phantom temperatures of 28°C, it took more than 4 hr to reach equilibrium with the environment.

**Table 3:** Phantom temperatures (mean ± SD) measured via optic fiber sensors and times to equilibrate with temperature inside a magnet bore at 3 T

Temperature inside magnet bore = 22.8 ± 0.6°C			
Sensor No.	Initial temperature	Final temperature	Time to equilibrate with the environment
OFS#1	28.4 ± 0.5°C	22.7 ± 0.6°C	4 hrs 08 min
OFS#2	28.3 ± 0.6°C	22.8 ± 0.5°C	4 hrs 02 min
OFS#3	28.3 ± 0.6°C	22.8 ± 0.6°C	4 hrs 07 min
OFS#4	28.2 ± 0.5°C	22.7 ± 0.6°C	4 hrs 11 min

Table 4 shows temperatures obtained by the diffusion coefficients and measured using four optic fiber temperature sensors in the phantom on one of 3 T MRI scanners. The measured temperature changes by the two methods agreed very well, with the difference between these two methods ranging from 6% to 9%.

**Table 4:** Temperatures obtained by diffusion coefficients and measured via optic fiber temperature sensors before and after high SAR image sequence on one of two same model Philips 3 T (T2w TSE and MRI system‐reported SAR value = 1.5 W kg^‐1^)

		Before high SAR image sequence	After high SAR image sequence	Temperature difference between before and after high SAR image sequence
Position #1	Mean diffusion coefficient [× 10^−3^ mm^2^ s^−1^]	2.1462	2.1553	
	Temperature obtained by mean diffusion coefficient [K]	295.41	295.57	0.16
	Temperature measured via temperature sensor [°C]	24.18	24.35	0.17
Position #2	Mean diffusion coefficient [× 10^−3^ mm^2^ s^−1^]	2.1473	2.1542	
	Temperature obtained by mean diffusion coefficient [K]	295.43	295.56	0.13
	Temperature measured via temperature sensor [°C]	24.01	24.15	0.14
Position #3	Mean diffusion coefficient [× 10^−3^ mm^2^ s^−1^]	2.1473	2.1533	
	Temperature obtained by mean diffusion coefficient [K]	295.43	295.54	0.11
	Temperature measured via temperature sensor [°C]	24.16	24.28	0.12
Position #4	Mean diffusion coefficient [× 10^−3^ mm^2^ s^−1^]	2.1484	2.1583	
	Temperature obtained by mean diffusion coefficient [K]	295.45	295.63	0.18
	Temperature measured via temperature sensor [°C]	24.21	24.38	0.17

The results of the measured high SAR values for each scanner are summarized in Table 5. For the GE 1.5 T MRI system, the MRI scanner‐reported SAR value was 1.58 W kg^‐1^ and the measured SAR value was 1.38 W kg^‐1^. For the Philips 1.5 T MRI scanner, the MRI system‐reported SAR value was 1.48 W kg^‐1^ and the measured value was 1.39 W kg^‐1^. For the Siemens 3 T MRI system, the reported SAR value was 2.5 W kg^‐1^ and the measured SAR value was 1.96 W kg^‐1^. For the two same model Philips 3 T MRI scanners, the reported SAR values were 1.5 W kg^‐1^ and the measured values were 1.94 and 1.96 W kg^‐1^. Percentage differences between the measured and reported SAR values on the GE 1.5 T, Philips 1.5 T, Siemens 3 T, and Philips 3 T were 13.5, 6.3, 24.2, 25.6, and 26.6% respectively.

**Table 5:** SAR measurement results using MR DTI from four MRI systems

Image sequence	GE 1.5 T	Philips 1.5 T	Siemens 3 T	Philips 3 T	Philips 3 T
	T1w TSE	T1w TSE	T1 TIRM	T2w TSE	T2w TSE
Temperature difference between before and after the high SAR image sequence [°C] (mean ± SD)	0.08 ± 0.01	0.07 ± 0.01	0.11 ± 0.01	0.18 ± 0.02	0.18 ± 0.02
Measured SAR value induced by a DTI scan [W kg^‐1^] (mean±SD)	0.11 ± 0.01	0.21 ± 0.02	0.48 ± 0.04	0.53 ± 0.05	0.51 ± 0.05
High SAR sequence scan time [sec]	168	169	186	320	320
MRI system‐reported SAR value [W kg^‐1^]	1.58	1.48	2.5	1.5	1.5
SAR value measured for the high SAR image sequence [W kg^‐1^] (mean ± SD)	1.38 ± 0.09	1.39 ± 0.12	1.96 ± 0.18	1.94 ± 0.18	1.96 ± 0.19
Percentage difference between measured and reported SAR values [%]^a^	−13.5	−6.3	−24.2	25.6	26.6

TSE, Turbo spin echo; TIRM, Turbo inversion recovery magnitude.

a The sign ‘‐’ means that measured SAR value was lower than the reported one.

## 4 Discussion

Obtaining an accurate SAR measurement during an MRI scan is challenging. The scanner‐reported whole‐body averaged SAR value can be significantly different compared to the actual one.^15^ Diffusion coefficients allowed us to quantify the relative SAR levels of different pulse sequences and compare the levels of different MRI scanners from various MR device manufacturers and different magnetic field strengths.

It took around four hours for the phantom to equilibrate with the room temperature (Table 3). On the other hand, the SAR measurement was done much faster. It meant that the effect of SAR heating would not go away during the time of the measurement, which was about 10 min.

Details of the SAR calibration procedure used by vendors are unknown to the end user. Table 5 shows that the SAR values calculated by the MRI systems were not reliable. Usually an MRI scanner‐reported SAR is larger than the actual SAR,^16^ but two 3 T units of the same model showed a higher measured SAR value than that reported by the system. An MRI system can produce a higher than expected power output. This could be caused by drift of the coil Q‐factor or a malfunction of the RF transmit coil (e.g., an increased B counter‐rotating RF component). These results suggest that different MR manufacturers may have different calibrations of SAR values. One limitation of this work is that a SAR measurement based on diffusion in a “torso phantom” is not the same as a human measurement, which will also include the effects of perfusion, and sample heterogeneity.

Our results confirm that some vendors’ SAR values may not be reliable, and can only be used as an approximate guide. Although our study was done using 1.5 and 3 T scanners, the same approach can be applied to higher B_0_ fields. The scanner‐independent SAR measurements described in this study using diffusion coefficients thus can play a significant role in estimating accurate SAR values as a standardized method. This study can give radiologists greater confidence when they scan patients clinically. In addition, this test can be used as a tool for quality assurance and the calibration of MRI systems.

## 5 Conclusions

We demonstrated that SAR values measured by quantification of the water diffusion coefficient can be used as a feasible alternative to that calculated by clinical MRI systems.
